# Specificity of Adaptive Immune Responses in Central Nervous System Health, Aging and Diseases

**DOI:** 10.3389/fnins.2021.806260

**Published:** 2022-01-20

**Authors:** Chiara Rickenbach, Christoph Gericke

**Affiliations:** Institute for Regenerative Medicine, University of Zurich, Schlieren, Switzerland

**Keywords:** adaptive immune system, neurodegeneration, Alzheimer’s disease, antigen presentation, epitope mapping, neuroimmunology, T cells

## Abstract

The field of neuroimmunology endorses the involvement of the adaptive immune system in central nervous system (CNS) health, disease, and aging. While immune cell trafficking into the CNS is highly regulated, small numbers of antigen-experienced lymphocytes can still enter the cerebrospinal fluid (CSF)-filled compartments for regular immune surveillance under homeostatic conditions. Meningeal lymphatics facilitate drainage of brain-derived antigens from the CSF to deep cervical lymph nodes to prime potential adaptive immune responses. During aging and CNS disorders, brain barriers and meningeal lymphatic functions are impaired, and immune cell trafficking and antigen efflux are altered. In this context, alterations in the immune cell repertoire of blood and CSF and T and B cells primed against CNS-derived autoantigens have been observed in various CNS disorders. However, for many diseases, a causal relationship between observed immune responses and neuropathological findings is lacking. Here, we review recent discoveries about the association between the adaptive immune system and CNS disorders such as autoimmune neuroinflammatory and neurodegenerative diseases. We focus on the current challenges in identifying specific T cell epitopes in CNS diseases and discuss the potential implications for future diagnostic and treatment options.

## Introduction

With recent advances in single-cell technologies, the field of neuroimmunology has adapted the concept of ‘CNS immune cell coexistence’ (Mundt et al., 2019a). CNS ‘immune privilege’ was historically defined as the complete lack of brain immune surveillance and attributed to the supposed absence of conventional lymphatic vessels and complete restriction of immune cell access into the brain parenchyma. Nowadays, it is widely acknowledged that immune privilege is limited to the brain parenchyma and that immune reactivity in the meninges and cerebrospinal fluid (CSF)-filled spaces is comparable to the periphery. Moreover, immune cell traffic at the blood–brain barrier (BBB) is tightly regulated but not entirely restricted. Finally, the recent re-discovery of dural lymphatic vessels, allowing drainage of immune cells and CNS-derived solutes to peripheral lymph nodes, further contributed to an updated view of CNS-immune cell interactions (Engelhardt and Ransohoff, 2005; Engelhardt et al., 2017; Mundt et al., 2019a). While the first line of defense involves cells of the innate immune system such as macrophages and dendritic cells (DCs) at CNS borders, and brain parenchyma-resident microglia, adaptive immune cells such as T and B lymphocytes provide CNS immune surveillance *via* specific antigen-directed responses (Waisman et al., 2015). In fact, the adaptive immune system constantly protects the CNS from potential threats in the steady state and is required for normal brain development and homeostasis (reviewed in Ellwardt et al., 2016).

However, the responses of the adaptive immune system in neurodegenerative diseases are not fully understood. We still know little about the antigen-specificity and interconnection between immune responses and CNS disease pathology. In this review, we first provide an overview of the neuro-immune interactions in the steady state during aging and neurodegeneration. Next, we focus on specific adaptive immune responses in the context of neurological disorders, such as Alzheimer’s disease (AD) and Parkinson’s disease (PD), and inflammatory autoimmune diseases. Finally, we discuss current developments, technological approaches and challenges in T cell epitope mapping to unravel epitopes that potentially spark autoimmune responses in the context of CNS diseases and their significance for potential innovations in diagnostics and treatments.

## Central Nervous System-Immune Interactions in the Steady State

The interconnection between the immune and nervous systems is fundamental during CNS health and disease. Several studies have shown that immune cells access the CNS for regular immune surveillance to preserve and maintain full cognitive function (Hickey et al., 1991; Engelhardt and Ransohoff, 2005; Gadani et al., 2012). However, the access to the brain parenchyma, which harbors the neurons and glial cells, is tightly regulated to prevent excessive immune responses, which might lead to collateral neuronal damage.

The CNS is surrounded by three layers of meninges: the dura mater, the arachnoid mater, and the pia mater. Modern *in vivo* imaging technologies re-discovered that the dura mater contains not only dense blood vessels but also lymphatic vessels, which drain CSF (Aspelund et al., 2015; Louveau et al., 2015). This dural lymphatic system, therefore, provides a major efflux pathway for CSF solutes such as CNS-derived antigens and immune cells to deep cervical lymph nodes together with formerly described routes along the olfactory nerve through the cribriform plate and other cranial nerves (Weller et al., 1992; Louveau et al., 2018; Ahn et al., 2019). Adjacent to the dura, the arachnoid mater separates the dural vessels from CSF-containing subarachnoid space. The pia mater separates the subarachnoid space from the glia limitans-coated brain parenchyma and perivascular compartments (Weller, 2005). A variety of immune cells that are crucial for brain immune surveillance, such as DCs, border-associated macrophages (BAMs), T cells, innate lymphoid cells (ILCs), neutrophils, and B cells, populate the meninges and other CSF-filled CNS boundaries such as perivascular spaces and the choroid plexus (Kivisäkk et al., 2003; Jordão et al., 2019; Van Hove et al., 2019). Meningeal DCs and BAMs act as antigen-presenting cells (APCs) and present local CNS-derived antigens to patrolling T cells, enabling effective brain immune surveillance (Rustenhoven et al., 2021). Therefore, the meninges are a critical site of communication between the CNS and the immune system (Rustenhoven et al., 2021). Meningeal myeloid cell pools, neutrophils, and B cells are supplied by hematopoietic cells from the skull and vertebral bone marrow (Brioschi et al., 2021; Cugurra et al., 2021; Schafflick et al., 2021). In addition, recent studies on meningeal B cells show that locally matured B cells educated by CNS-derived antigens are subject to tolerance mechanisms to negatively select CNS-reactive B cells (Brioschi et al., 2021; Wang Y. et al., 2021).

The glia limitans is composed of astrocyte processes and parenchymal basal membrane. At the site of brain capillaries, the endothelial basal membrane is fused with parenchymal basal membrane forming a BBB with low permeability for fluids and solutes. At post-capillary venules, the basal membranes of endothelial cells and glia limitans are separated by a perivascular space which is inhabited by antigen-presenting cells such as DCs and BAMs, providing a site for presentation of CNS-derived epitopes (Quintana et al., 2015; Van Hove et al., 2019).

The choroid plexus secretes the CSF, the fluid that fills the cerebral ventricles, subarachnoid spaces, and perivascular spaces. The choroid plexus epithelium is a blood–CSF barrier (BCSFB) that regulates the entry of immune cells into the CNS. DCs, BAMs, and microglia inhabit the choroid plexus stroma, and immune cells such as T cells can routinely enter the CSF through the choroid plexus epithelium (Kivisäkk et al., 2003; Engelhardt and Ransohoff, 2005; Engelhardt et al., 2017; Van Hove et al., 2019; Dani et al., 2021). Indeed, T cells, B cells, DCs, and monocytes are present in the healthy CSF (Kivisäkk et al., 2003; Schafflick et al., 2020). Beside the previously mentioned CSF efflux pathways, CNS antigens drain to cervical lymph nodes *via* the glymphatic (glial-lymphatic) pathway (Engelhardt et al., 2016). The CSF flows from the perivascular space of leptomeningeal arteries to the brain interstitium, where it becomes part of the interstitial fluid (ISF). ISF exits *via* venous perivascular space and clears brain-derived solutes, including amyloid-β (Aβ) peptides, into meningeal lymphatic vessels (Iliff et al., 2012, 2013; Rasmussen et al., 2018). As immune cells can reach the CSF through subarachnoid veins and choroid plexus epithelium, the immune accessibility of the meninges and CSF-filled spaces is comparable to those of the periphery.

Within the brain parenchyma, microglia are the most common immune cells. Microglia are brain phagocytes, which are involved in synaptic pruning and plasticity and support normal brain development and immune surveillance in the steady state (Paolicelli et al., 2011; Cunningham et al., 2013; Salter and Beggs, 2014). Moreover, the brain parenchyma is inhabited by low numbers of adaptive immune cells, such as CD4 and CD8 T cells (Smolders et al., 2013, 2018; Herich et al., 2019; Pasciuto et al., 2020).

**FIGURE 1:**
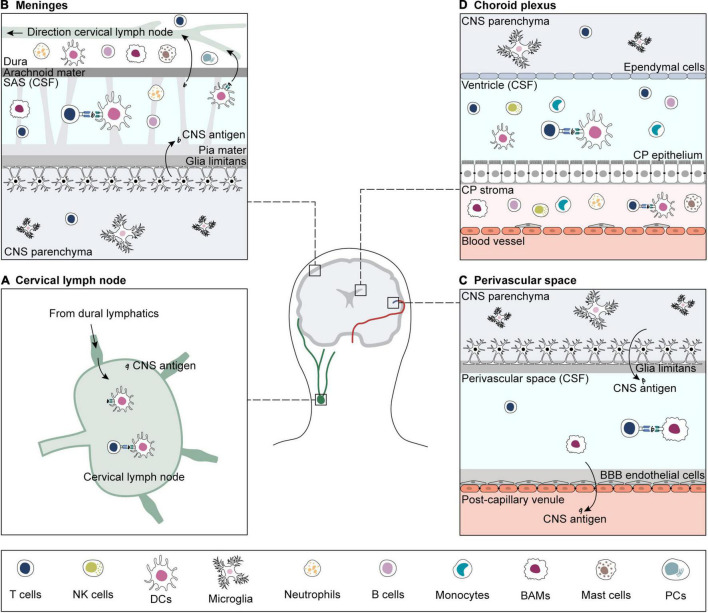
Sites of antigen presentation of CNS-derived epitopes. CNS-derived antigens from the brain parenchyma drain to deep cervical lymph nodes and prime matching T cells **(A)**. For reactivation, CNS-reactive T cells extravasate from blood vessels in search for their cognate antigen on APCs in sub-arachnoid space (SAS) **(B)**, perivascular space **(C)** and choroid plexus (CP) **(D)** (Greter et al., 2005; Bartholomäus et al., 2009; Reboldi et al., 2009; Schläger et al., 2016; Mundt et al., 2019b; Rustenhoven et al., 2021). If re-activation is successful, CNS-reactive T cells infiltrate the brain parenchyma. CNS borders are inhabited by a variety of immune cells, including T and B cells, B plasma cells (PCs), NK cells, border-associated macrophages (BAMs), dendritic cells (DCs), monocytes, and granulocytes (neutrophils and mast cells) (Goldmann et al., 2016; Jordão et al., 2019; Mundt et al., 2019b; Van Hove et al., 2019; Fitzpatrick et al., 2020; Schafflick et al., 2020, 2021; Brioschi et al., 2021; Dani et al., 2021; Rustenhoven et al., 2021). The brain parenchyma contains microglia and small numbers of memory T cells (Smolders et al., 2013, 2018; Herich et al., 2019; Pasciuto et al., 2020).

The priming location of CNS-reactive T cells has long been debated. As naïve T cells cannot enter the brain *via* the BBB vascular endothelium and BCSFB, they are thought to be primed in peripheral CNS-draining lymph nodes (Korn and Kallies, 2017; Mundt et al., 2019a). Primed CNS-reactive T cells can cross the BBB endothelium and the walls of subpial venules, but to cross the glia limitans, they need to be re-activated by local APCs loaded with their cognate antigen. Sites of antigen presentation for re-stimulation and T cell entry into the brain are the perivascular space, the meninges, and the choroid plexus (Figure 1; Greter et al., 2005; Bartholomäus et al., 2009; Reboldi et al., 2009; Schläger et al., 2016; Mundt et al., 2019b; Rustenhoven et al., 2021).

Evidence of CNS surveillance by adaptive immune cells was provided in the context of treatment of multiple sclerosis (MS) and Crohn’s disease patients with natalizumab, a monoclonal antibody against α4 integrins that prevents lymphocyte traffic in the CNS. A small fraction of patients developed progressive multifocal leukoencephalopathy (PML), a deadly opportunistic CNS infection caused by re-activation of the latent John Cunningham (JC) polyomavirus (Berger et al., 2015). Immunocompromised patients are susceptible to other opportunistic infections of the CNS, such as herpes encephalitis caused by herpes simplex viruses and toxoplasmosis (Tan et al., 2012; Schmidt et al., 2013). Such findings highlight the crucial role of the adaptive immune system in protecting the brain from infections. Interestingly, adaptive immune cells also contribute to the maintenance of CNS homeostasis. Neurogenesis was shown to depend, among other factors, on the adaptive immune system: T cell deficiency leads to defective hippocampal neurogenesis and cognitive deficits (Ziv et al., 2006). Thus, adaptive immune cells in the CNS have multifaceted roles, ranging from protective and homeostatic functions to damaging and disease-promoting roles, as we will discuss in later paragraphs.

## Central Nervous System-Immune Interactions During Aging and Neurodegeneration

### Aging of the Adaptive Immune System

As age is the most prominent risk factor for neurodegenerative diseases, it is important to first address the changes that the immune system and CNS already experience just during aging (Deleidi et al., 2015). Aging is accompanied by a functional decline of the immune system and CNS. The systemic pro-inflammatory environment that is created during aging is defined as “inflammaging.” Inflammaging is caused by the secretion of pro-inflammatory cytokines and chemokines (IL-1, IL-6, TNFα) by immunosenescent cells as part of a senescence-associated secretory phenotype (SASP) (Franceschi et al., 2000; Freund et al., 2010). Chronic viral infections and antigen exposure cause overstimulation and dysfunction of the immune system, thus promoting inflammaging (Franceschi et al., 2000, 2007; Freund et al., 2010). Immune senescence and inflammaging contribute to changes in peripheral immune cell signature occurring during aging.

Aging causes changes in peripheral T cell composition due to age-dependent thymic involution (Goronzy and Weyand, 2019). As T cells are not produced *de novo* in the thymus in adults, the production of naïve T cells relies on the homeostatic proliferation of existing naïve T cell pools. Impaired homeostatic proliferation, differentiation to memory cells upon chronic antigen exposure, and failed quiescence together lead to decreased naïve T cell pools in the elderly (Goronzy and Weyand, 2019; Carrasco et al., 2021). In addition, thymic involution contributes to the generation and accumulation of autoreactive T cells in peripheral organs, including the brain, and a higher risk of autoimmune diseases (Coder et al., 2015). Chronic viral infections in the older age, most commonly, e.g., cytomegalovirus (CMV) infections, trigger an expansion of dysfunctional antigen-specific T cells as well as exhaustion and senescence of naïve and memory T cells (Franceschi et al., 2007). Moreover, in older humans, the numbers of effector memory T cells that re-express CD45RA (TEMRA) increase (Quinn et al., 2018). TEMRA cells display senescent-like features, including a SASP phenotype and higher cytokine production (Mahnke et al., 2013; Goronzy and Weyand, 2019). Mogilenko and colleagues recently reported the emergence of a subset of clonally expanded, age-associated, granzyme K-expressing (GZMK+) CD8 T cells, displaying markers of cell exhaustion and tissue homing. GZMK+ CD8 T cells contribute to inflammaging by promoting SASP in senescent cells (Mogilenko et al., 2020). In parallel, the frequency of regulatory T cells (Tregs) is increased with age. Still, their regulatory function is equivalent in the young and elderly and may contribute to the decline in adaptive immune responses observed during aging (Gregg et al., 2005).

Along with changes in relative immune cell composition, T cells also experience phenotype alterations during aging. Functional defects in T cells during aging could be dependent on both changes in population composition and altered cellular phenotype as well as activity (Srinivasan et al., 2021). CD4 T cells exhibit age-related impairments in the formation of the immunological synapse, reduced homing to secondary lymphoid organs, and reduced TCR sensitivity and signaling strength (Garcia and Miller, 2011; Li et al., 2012; Richner et al., 2015; Srinivasan et al., 2021). To ensure efficient immune responses, both TCR diversity and population size are essential. In the elderly, the diversity of the TCR repertoire decreases, and T cell clonality increases together with imbalances in clonal size, especially in the naïve compartment (Qi et al., 2014). However, it is unclear whether these changes have a functional significance in establishing a successful immune response. An important consequence of decreased T cell function in older age is the reduced ability to form an immune memory for novel antigens, and thus the increased susceptibility to certain infections and reduced effectiveness of vaccines in older people (Erickson and Banks, 2019).

### The Effect of Aging on Central Nervous System Barriers and the Central Nervous System-Immune Interface

Specifically in the CNS, inflammaging causes activation of brain cells by altering microglia phenotype and reactivity, thus promoting low-grade inflammation in the brain (Von Bernhardi et al., 2010). In a vicious cycle, brain inflammation modulates the immune system by promoting immunosenescence and inflammaging (Deleidi et al., 2015). This highlights the co-dependency of the two systems in both health and aging. Similar to the periphery, normal brain aging is characterized by an inflammatory environment and increased secretion of pro-inflammatory cytokines (Von Bernhardi et al., 2010). Resident microglial cells become senescent and activated in aged brains accompanied by changes in morphology and distribution (Streit et al., 2004; Wong, 2013). Similarly, aged microglia in mice acquire an activated immunophenotype (Mrdjen et al., 2018). Increased microglial activation contributes to brain inflammaging and the risk of developing neurodegenerative diseases (Wong, 2013; Deleidi et al., 2015).

During aging, meningeal lymphatics and therefore the meningeal CNS-immune interface experience functional decline. CNS lymphatic drainage to cervical lymph nodes *via* the glymphatic system and meningeal lymphatics is impaired during aging (Kress et al., 2014; Da Mesquita et al., 2018). Since meningeal lymphatic vessels facilitate the efflux of macromolecules such as Aβ peptides from interstitial fluids and CSF, any disturbance favors the deposition of potentially neurotoxic substances in the brain. In addition, the size of the lymphatic vessels in the meninges and the coverage of the brain are reduced in older age, so that CSF outflow is impaired and CSF production is reduced. These aging-associated perturbations of CNS drainage may be an important factor in the development of neurodegenerative diseases (Ma et al., 2017; Da Mesquita et al., 2018; Ahn et al., 2019; Liu et al., 2020). Moreover, a dysfunctional meningeal lymphatic system might also affect the traffic of immune cells to cervical lymph nodes (Ahn et al., 2019).

To date, it is not known what causes the alteration of the immune cell composition in the aged brain. The functional decline of CNS clearance systems and parallel BBB breakdown during aging are suggested to be involved in the increased infiltration of lymphocytes into the brain (Montagne et al., 2015). Increased T cell invasion into the sub-ventricular zone of older brains was reported, along with the emergence of a reactive microglia subset as well as modified composition of BAM and DC subsets in geriatric mice (Mrdjen et al., 2018; Dulken et al., 2019). CD8 T cell accumulation in the brain was shown to impair neurogenesis, drive axon degeneration, and contribute to cognitive and motor decline (Dulken et al., 2019; Groh et al., 2021).

### Change in Central Nervous System-Barriers and Central Nervous System-Immune Interface in Neurodegenerative Diseases

Impaired meningeal lymphatic function is involved in the pathophysiology of neurodegenerative diseases and accelerates cognitive decline and neuroinflammation (das Neves et al., 2021). In brain β-amyloidosis, the meningeal lymphatic system is defective, leading to increased Aβ deposition and brain pathology, microglial inflammatory response, and cognition (Da Mesquita et al., 2018, 2021). Conversely, enhancing meningeal lymphatic function *via* injection of vascular endothelial growth factor (VEGF) improved the outcome of Aβ-passive immunotherapy (Da Mesquita et al., 2021). Dysfunctional lymphatic drainage was reported in human idiopathic PD (Ding et al., 2021). Similarly, in MS, decreasing lymphatic drainage into deep cervical lymph nodes improved the symptoms of experimental autoimmune encephalomyelitis (EAE) mouse model (Furtado et al., 2008; Louveau et al., 2018). Dysfunction of BBB starts in the early stages of AD and other neurodegenerative diseases such as PD, MS, and amyotrophic lateral sclerosis (ALS), and it is associated with reduced cerebral blood flow, increased permeability, and microbleeds (Sweeney et al., 2018). BBB dysfunctions contribute to increased infiltration of inflammatory cells in the inflamed CNS. As we describe below, increased infiltration of DCs, monocytes, and T cells in the brain parenchyma has been reported in several age-related neurodegenerative diseases, such as AD and PD, and in MS.

## Adaptive Immune Responses in Neurodegenerative Disorders

### Alzheimer’s Disease

#### β-Amyloidosis as Pathophysiological Hallmark of Alzheimer’s Disease

Alzheimer’s disease is a neurodegenerative disorder characterized by accumulation and deposition of misfolded Aβ in the form of β-amyloid plaques and aggregation of abnormally phosphorylated tau, as well as neurodegeneration, ultimately leading to cognitive decline (Jack et al., 2018). Aβ monomers assemble into soluble, low molecular weight oligomers, which spread throughout the brain and subsequently aggregate into insoluble Aβ fibrils, gradually forming β-amyloid plaques (Chen et al., 2017). Tau aggregation into intracellular neurofibrillary tangles (NFT) is fostered by various post-translational modifications such as hyperphosphorylations (Querfurth and Laferla, 2010; Wesseling et al., 2020). The “amyloid cascade hypothesis” states that Aβ accumulation, aggregation, and deposition are primary pathological events of AD (Hardy and Higgins, 1992; Selkoe and Hardy, 2016). In fact, the temporal development of AD-associated biomarker abnormalities begins with Aβ-related changes in CSF and cerebral cortex, decades before the onset of detectable neuropsychological abnormalities (Buchhave et al., 2012; Jack et al., 2013).

Imbalances in the production and clearance of Aβ from the brain are suspected of favoring the pathological aggregation process (Zlokovic, 2011; Iliff et al., 2012). Since ISF-CSF flow contributes to Aβ clearance, Aβ and tau are retrievable in both CSF and peripheral blood (Seubert et al., 1992; Shibata et al., 2000; Weller et al., 2008; Zlokovic, 2011; Iliff et al., 2012). In the brain, Aβ is locally degraded by enzymes in brain-resident cell types, including microglia and astrocytes, which contribute to a reduction of β-amyloid load in the brain. As APP is also expressed in other organs and tissues, peripherally produced Aβ contributes to the total Aβ pool (Roher et al., 2009). In the periphery, Aβ is cleared *via* phagocytosis and endocytosis by various cell types, including monocytes, macrophages, and neutrophils (Bradshaw et al., 2013; Wang et al., 2017). Amyloid burden in the brain is further reduced by sequestration of soluble Aβ in the circulation by anti-Aβ antibodies and LRP1 *via* a peripheral ‘sink’ effect (DeMattos et al., 2002; Sagare et al., 2007).

#### Adaptive Immune Responses in Alzheimer’s Disease

**FIGURE 2:**
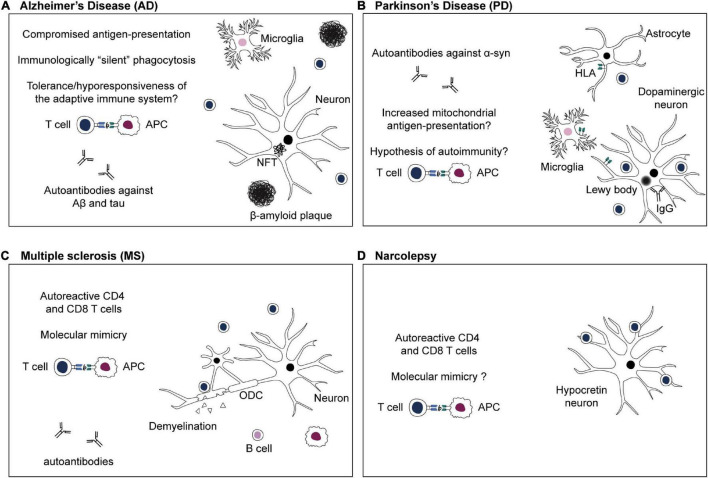
Adaptive immune system in CNS disorders. **(A)** Alzheimer’s disease: the antigenic targets of infiltrating T cells in the brain during β-amyloidosis and tau pathology are not known yet. Efficient phagocytosis and antigen presentation are compromised in AD (Gu et al., 2016; Gericke et al., 2020). Phagocytosis of Aβ aggregates might happen in an immunologically “silent” manner, or, chronic exposure to Aβ-derived self-peptides and tolerance mechanisms might lead to a hyporesponsiveness of the adaptive immune system (Monsonego et al., 2001; Ferretti et al., 2016). **(B)** Parkinson’s disease: targeted antigen-specific T cell infiltration in the brain of PD patients is thought to be due to expression of HLA class II or class I by microglia, astrocytes, and dopaminergic neurons, respectively (Imamura et al., 2003; Cebrián et al., 2014; Rostami et al., 2020). Moreover, defects in PD-associated proteins PINK1 and parkin might increase presentation of mitochondrial antigens, leading to autoimmunity (Matheoud et al., 2016). **(C)** Multiple sclerosis: numerous studies emphasize the crucial role of autoreactive CD4 and CD8 T cells in MS (Dendrou et al., 2015). Autoreactive T cells infiltrate the CNS and cause demyelination as well as oligodendrocytes (ODC) and neuroaxonal damage (Dendrou et al., 2015). Autoreactive T cells might be activated in the periphery *via* recognition of viral antigens that are similar to a self-peptide *via* molecular mimicry (Sospedra and Martin, 2005; Dendrou et al., 2015). **(D)** Narcolepsy: autoreactive CD4 and CD8 T cells target antigens of hypocretin neurons and are thought to be responsible for neuronal damage (Latorre et al., 2018; Luo et al., 2018). The existence of T cells cross-reacting to influenza epitopes and hypocretin peptides, and the role of molecular mimicry in narcolepsy are still a subject of debate (Latorre et al., 2018: Luo et al., 2018).

The first evidence for the involvement of the adaptive immune system in AD originates from the discovery of increased CD4 and CD8 T cell numbers in the brains of AD patients (Figure 2A; Itagaki et al., 1988; Togo et al., 2002). Until today, it is not fully understood whether T cell extravasation into the CNS results from AD pathology-related degeneration of brain barriers and microbleeds or if T cells are specifically recruited to the brain by recognizing CNS-derived antigens. Studies in mouse models of β-amyloidosis and *post mortem* brain material of AD patients showed that while micro-hemorrhages were contributing to unspecific T cell extravasation, specific T cell infiltration into the brain parenchyma was mostly associated with β-amyloidosis and tau pathology (Ferretti et al., 2016; Laurent et al., 2017; Merlini et al., 2018). However, those T cells were mainly not co-localized with β-amyloid plaques and NFTs, suggesting that cerebral β-amyloid and tau load is necessary but not sufficient for T cell invasion (Togo et al., 2002; Ferretti et al., 2016; Laurent et al., 2017; Merlini et al., 2018). T cell antigen specificity is still unknown in this case; however, epitopes with antigenic potential might be hidden within the 3D structure of β-amyloid plaques and NFTs and released upon disassembly or digestion of aggregates. Alternatively, novel T cell antigens, so-called neo-epitopes, might be formed by splicing mechanisms during processing within APCs (Faridi et al., 2018). Modern technological approaches such as high-throughput single-cell epitope mapping to identify these potential neo-epitopes are in high demand and will be discussed later.

Further evidence for the involvement of adaptive immunity in AD comes from genome-wide association studies that found several genetic risk factors among major histocompatibility complex alleles (MHC, human leukocyte antigen/HLA in humans) (HLA-DQA*01:02, HLA-DRB1*15:01, HLA-DQB1*06:02) and other genes associated with phagocytosis and antigen presentation (*TREM2, CD33, MS4A6A*) (Kunkle et al., 2019). Notably, the immune receptor TREM2 is required for microglia responses, including accumulation in proximity to Aβ deposition and phagocytosis of Aβ plaques (Ulrich et al., 2014; Yeh et al., 2016). Wang and colleagues showed that treatment with agonist antibody against TREM2 promotes microglia activation and induces phagocytosis of Aβ in mouse models of β-amyloidosis, reducing the toxicity of Aβ plaques and neuronal damage (Wang S. et al., 2020). Antigen-dependent T cell infiltration in the brain during AD requires successful antigen presentation and T cell re-activation at the CNS borders. As microglia are located in proximity to β-amyloid plaques and phagocyte Aβ aggregates, they may serve as local APCs for re-activation of Aβ-reactive T cells (Perlmutter et al., 1992; Monsonego and Weiner, 2003; Lee and Landreth, 2010; Mittal et al., 2019). However, phagocytosis and antigen presentation are compromised in AD (Gu et al., 2016). β-amyloidosis was reported to interfere with CNS antigen presentation and was linked to reduced T cell activation in brains of AD mouse models of β-amyloidosis (Ferretti et al., 2016; Gericke et al., 2020). Specifically, Aβ oligomers were shown to inhibit antigen presentation by reducing MHC-II surface expression on APCs and T cell activation in mice with β-amyloidosis (Gericke et al., 2020). Thus, Aβ phagocytosis might occur in an immunologically “silent” manner, without leading to an adaptive immune response (Figure 2A; Fitzner et al., 2011; Ferretti et al., 2016). Alternatively, efficient phagocytosis and antigen presentation might be prevented by tolerogenic responses. Infiltrating T cells in brains with Aβ burden lack cytokine signaling (e.g., IFNγ production) and effector function (Ferretti et al., 2016). This supports the hypothesis that T cells might have a regulatory function upon infiltration in the CNS in AD. In the long term, it might lead to a decline in the ability of microglia and macrophages to clear Aβ from the CNS and immune evasion, driving an increased formation of Aβ aggregates (Schroder et al., 2004).

#### β-Amyloid-Directed Autoreactive Immune Responses

Although antigens responsible for T cell infiltration in the brain during AD have not been identified, T cells with reactivities toward Aβ-derived epitopes were detected in the blood of middle-aged, elderly, and AD patients (Table 1; Monsonego et al., 2003). Restriction of these T cells to HLA-DR demonstrated that Aβ-specific T cells are activated by APCs presenting Aβ-derived epitopes (Zota et al., 2009). However, no difference in reactivity between healthy subjects and AD patients was observed, suggesting some kind of tolerance mechanism that prevents an overt autoimmune response against physiological Aβ (Monsonego et al., 2003; Dhanwani et al., 2020). This hyporesponsiveness of the adaptive immune system to Aβ-derived epitopes might be due to chronic exposure to Aβ peptide and concomitant tolerance mechanisms. As the immune system is exposed to Aβ peptide before deposition as Aβ plaques, Aβ-specific T cells may be negatively selected in the thymus during early development or be subject to peripheral tolerance. In a β-amyloidosis mouse model, a decreased immune response to Aβ peptide is also observed in the context of humoral immunity. Antibody production is partially restored by stimulating T cells with Aβ peptide coupled to BSA, hinting that the observed hyporesponsiveness is due to defective T cell help (Monsonego et al., 2001). The adverse involvement of lymphocytes in AD was further demonstrated by a study showing that mice lacking functional T and B cells (PSAPP, *Rag2*^–/–^) have reduced β-amyloid pathology in a model of β-amyloidosis (Späni et al., 2015). However, another study employing a different mouse model lacking T, B, and NK cells (5xfAD, *Rag2*^–/–^ *Il2rg*^–/–^), showed accelerated β-amyloid pathogenesis and increased neuroinflammation (Marsh et al., 2016). Overall, the role of T cells in AD needs to be further elucidated.

**TABLE 1:** T cell epitopes associated with AD, PD, MS, and narcolepsy.

Epitope	Species	HLA allele	References
**Alzheimer’s disease**			
Aβ	Human	HLA-DR15 HLA-DR3 HLA-DR4 HLA-DR1 HLA-DR13	Monsonego et al., 2003; Zota et al., 2009
**Parkinson’s disease**			
α-Syn	Human	–	Sulzer et al., 2017; Lindestam Arlehamn et al., 2020
**Multiple sclerosis**			
MBP	Human	HLA-DR15, HLA-DR4	Olsson et al., 1992; Cao et al., 2015; Wang J. et al., 2020
PLP	Human	HLA-DR15, HLA-DR4	Correale et al., 1995; Cao et al., 2015; Wang J. et al., 2020
MOG	Human	HLA-DR15, HLA-DR4	Wallstrom et al., 1998; Cao et al., 2015; Wang J. et al., 2020
RASGRP2	Human	HLA-DR15	Jelcic et al., 2018
GDP-L-fucose	Human	HLA-DRB3*02:02	Planas et al., 2018
BHRF1	EBV	HLA-DR15	Wang J. et al., 2020
BPLF1	EBV	HLA-DR15	Wang J. et al., 2020
Sugar-binding protein	*Akkermansia muciniphila*	HLA-DR15	Wang J. et al., 2020
Peptidase M50	*Akkermansia muciniphila*	HLA-DR15	Wang J. et al., 2020
*N*-Acetyltransferase	*Akkermansia muciniphila*	HLA-DR15	Wang J. et al., 2020
EBNA1	EBV	–	Lünemann et al., 2008
**Narcolepsy**			
HCRT	Human	HLA-DQB1*06:02	Latorre et al., 2018
TRIB2	Human	HLA-DQB1*06:02	Latorre et al., 2018
HA	Influenza A virus subtype H1N1	HLA-DQB1*06:02	Luo et al., 2018

The discovery of human autoantibodies against Aβ produced by immortalized B cells obtained from an AD patient proved successful priming of B cells against aggregated Aβ species in humans (Gaskin et al., 1993). Autoantibodies against Aβ and tau occur naturally in blood and CSF of AD patients (Geylis et al., 2005; Kellner et al., 2009; Bartos et al., 2012; Maftei et al., 2013). A potential neuroprotective role and an association between levels of autoantibodies and AD progression have been debated (Hyman et al., 2001; Britschgi et al., 2009; Dodel et al., 2011; Mengel et al., 2013; Söllvander et al., 2015). Several immunotherapies have been developed to prevent and reduce β-amyloid plaque deposition by exploiting the humoral immune response. In a mouse model of β-amyloidosis, active Aβ immunization reduced the formation of β-amyloid plaques and memory loss (Schenk et al., 1999; Janus et al., 2000; Morgan et al., 2000). In humans, cognitive decline was reduced in patients who developed an antibody response against Aβ after active immunization, with beneficial antibody titers persisting for several years after initial vaccination (Hock et al., 2003; Vellas et al., 2009). However, the human trial was discontinued because of life-threatening T cell-associated meningoencephalitis in 6% of the patients (Orgogozo et al., 2003). Alternatively, passive immunotherapy with anti-Aβ antibodies, particularly antibodies that react against the aggregated state of Aβ, also reduces β-amyloid plaque load and slows cognitive decline, confirming that humoral immune responses may be the basis of future therapies reducing the β-amyloid burden and ameliorating AD symptoms (Sevigny et al., 2016; Mintun et al., 2021).

#### Single-Cell Immunophenotyping of Disease-Associated T and B Cells

In recent years, immunophenotyping studies with high-dimensional flow and mass cytometry, as well as single-cell RNA sequencing, tried to identify disease-specific immune cell signatures. Several studies reported AD-associated changes in peripheral immune cell compositions in AD patients, such as increase of memory CD4 T cells and activated CD8 and CD4 T cells, and a parallel decrease of CD4 naïve T cells, compared to healthy control subjects (Larbi et al., 2009; Pellicanò et al., 2012; Lueg et al., 2015). Recently, multi-dimensional mass cytometry revealed increased proportions of CD8 TEMRA cells in the blood and CSF of AD patients compared to healthy controls (Gate et al., 2020). A subsequent study including β-amyloid-PET-characterized pre-symptomatic AD patients revealed systemic changes within T and B cell populations concomitant to β-amyloid accumulation. Relative abundances of peripheral CD8 TEMRA cells are increased, and naïve CD8 T cells are decreased, already in cognitively healthy β-amyloid-positive subjects compared to β-amyloid-negative healthy controls subjects (Gericke et al., 2021). In parallel, numbers of peripheral naïve B cells are reduced, and antibody-secreting and memory B cells are increased in β-amyloid-positive healthy controls (Gericke et al., 2021). Therefore, systemic changes within the T and B cell compartments are detectable during early cerebral β-amyloid accumulation in pre-clinical AD stages. Overall, these studies showing immune cell signatures that correlate with AD emphasize the role of the adaptive immune system in AD.

#### Viral Infections and Alzheimer’s Disease

The antimicrobial protection hypothesis states that Aβ deposition is induced as part of an innate immune response against pathogens. Aβ fibrils entrap invading pathogens and promote neuroinflammation and clearance of the infection (Moir et al., 2018). During AD, this pathway is chronically activated and leads to detrimental inflammation and neurodegeneration. This hypothesis is supported by reports showing that Aβ acts as an antimicrobial peptide *in vitro* and in a mouse model of β-amyloidosis, protecting against viral infections (White et al., 2014; Bourgade et al., 2015; Kumar et al., 2016; Eimer et al., 2018). Accordingly, increased microbial burden and *Herpesviridae* infections accelerate β-amyloidosis, inflammation, and AD progression in mice with β-amyloidosis (Eimer et al., 2018). In humans, herpesviruses are associated with Aβ plaques and drive AD development (Jamieson et al., 1991; Wozniak et al., 2005, 2009; Lurain et al., 2014; Itzhaki et al., 2020). Another potential link between viral infections and AD is provided by the discovery of clonally expanded CD8 TEMRA cells in the CSF of AD patients specific for EBV-derived epitopes (Gate et al., 2020). However, this notion is challenged by analysis of viral background infections by herpesviruses, demonstrating that pre-symptomatic β-amyloidosis causes alterations of B and T cells populations independently of latent viral infections (Gericke et al., 2021). Therefore, a causal relationship between viral infections and adaptive immune response in AD still has to be determined.

### Parkinson’s Disease

Parkinson’s disease is a neurodegenerative disorder characterized by loss of dopaminergic neurons in the substantia nigra pars compacta of the midbrain, leading to motor symptoms and cognitive impairment (Kalia and Lang, 2015). The hallmark of PD is Lewy pathology, which is the presence of intracellular aggregates of abnormally folded α-synuclein (α-syn) in the neuronal cell body (Lewy bodies) and neurites (Kalia and Lang, 2015). Lewy pathology appears first in the brainstem and progressively spreads to the substantia nigra and cortex, supposedly *via* cell-to-cell transmission (Braak et al., 2004; Kordower et al., 2008; Li et al., 2008; Visanji et al., 2014). Extracellular α-syn can be taken up by neurons, astrocytes, and microglia for degradation, but it is also present in the ISF (Lee et al., 2008; Emmanouilidou et al., 2011). BBB dysfunction in PD patients leads to increased levels of α-syn in the CSF and plasma (Borghi et al., 2000; El-Agnaf et al., 2003; Kortekaas et al., 2005; Ham et al., 2014; Janelidze et al., 2015).

A role for the adaptive immune system in PD was suggested due to the observation of infiltrating T cells in PD brains and IgG on dopaminergic neurons and Lewy bodies (Figure 2B; Orr et al., 2005; Brochard et al., 2009). Moreover, autoantibodies against α-syn, which can block α-syn aggregation, have been detected in the blood and CSF of PD patients (McGeer et al., 1988a; Papachroni et al., 2007; Shalash et al., 2017; Li et al., 2019). Another indication of the importance of the adaptive immune system is elevated expression levels of HLA-DR on antigen-presenting cells in the CNS of individuals with PD, as well as alleles of the HLA locus that have been associated with PD risk (McGeer et al., 1988b; Fiszer et al., 1994; Hamza et al., 2010; Wissemann et al., 2013). Immunophenotyping studies showed that an altered composition of peripheral immune cells accompanies PD disease. Specifically, in blood and CSF of PD patients, proportions of naïve T cells are reduced, while activated and terminally differentiated T cells are increased in the CSF of PD patients compared to healthy elderly control subjects (Fiszer et al., 1994; Saunders et al., 2012; Schröder et al., 2018; Wang P. et al., 2021). Moreover, clonally expanded T cells are present in the blood and CSF of PD patients (Wang P. et al., 2021). Furthermore, a shift toward a more pro-inflammatory peripheral immune response was reported in PD patients (Thome et al., 2021). Concomitant with changes in immune cell populations, T cells reactive against α-syn-derived epitopes have been reported in pre-clinical and early-stage PD patients (Sulzer et al., 2017; Lindestam Arlehamn et al., 2020). CD4 T cell reactivity against α-syn epitopes increases with age in PD patients but not in healthy controls (Table 1; Lindestam Arlehamn et al., 2020). TCR analysis of α-syn-specific T cells revealed a diverse, patient-specific TCR repertoire (Singhania et al., 2021). Overall, these findings highlight a possible role for antigen-specific T cells in PD pathogenesis. However, a causal relationship between reactions of the adaptive immune system and the loss of dopaminergic neurons occurring in PD has not been established.

Similarly to AD, T cells located in the vicinity of dopaminergic neurons suggest a targeted antigen-specific T cell infiltration in the brain, rather than passive extravasation due to loss of BBB functionality (Brochard et al., 2009). Presentation of α-syn-derived epitopes and antigen-specific T cell infiltration into the brain parenchyma are also postulated because increased levels of HLA class II-expressing microglia can be found in the substantia nigra of severe PD patients compared to healthy controls and patients with mild PD. Moreover, expression of HLA class I on dopaminergic neurons in the substantia nigra renders them vulnerable to T cell-mediated neurodegeneration (Imamura et al., 2003; Cebrián et al., 2014). Supporting this hypothesis, mice lacking MHC-II are partially protected from dopaminergic neuron loss in a model of PD (Martin et al., 2016). As additional APC in the context of PD, astrocytes were shown to activate T cells *via* MHC-II in PD brains and express co-stimulatory molecules (CD80, CD86, and CD40), MHC-I, and MHC-II (Rostami et al., 2020). Moreover, Matheoud et al. (2016) elucidated the role of PINK1 and parkin, two PD-related proteins whose mutations cause early onset of the disease, in repressing mitochondrial antigen presentation (MitAP) (Kalia and Lang, 2015). In MitAP, mitochondrial autoantigens are transported to the lysosome *via* mitochondrial-derived vesicles and presented at the cell surface on MHC-I molecules. Defects in PINK1 and parkin might lead to an increased presentation of mitochondrial autoantigens and trigger autoimmunity. Although these studies suggest a crucial role of T cells in the loss of dopaminergic neurons during PD, the hypothesis of autoimmunity in PD still has to be investigated.

### Central Nervous System Autoimmune Diseases: Multiple Sclerosis and Narcolepsy

Aberrant immune responses directed against CNS self-antigens may lead to severe autoimmune diseases. In the following section, we will discuss MS, the most widespread CNS inflammatory disease caused by autoreactive immune cell responses, and narcolepsy, a well-studied example of a neurodegenerative disease mediated by autoimmunity.

### Multiple Sclerosis

Multiple sclerosis is the most common autoimmune disorder of the CNS. The disease has multifactorial origins and is characterized by infiltration of immune cells such as T cells, B cells, and myeloid cells into the brain and spinal cord, causing neuroinflammation, demyelination, and neuroaxonal degeneration (Dendrou et al., 2015). In particular, autoreactive CD4 T cells and, to a lesser extent, CD8 T cells are involved in MS pathogenesis, infiltrating the CNS and showing specificity for CNS autoantigen sources such as myelin basic protein (MBP), proteolipid protein (PLP), and myelin oligodendrocyte glycoprotein (MOG) (Table 1; Olsson et al., 1992; Correale et al., 1995; Wallstrom et al., 1998). Since CD4 T cells are HLA-class II restricted and parenchymal microglia express just low levels of class II HLA, myelin-autoreactive CD4 T cells are re-activated by DCs in the leptomeninges and perivascular space (Greter et al., 2005; Mundt et al., 2019b).

Genetic studies associated alleles of the HLA-DR15 haplotype (DRB1*1501, DRB5*0101, DQA1*0102/DQB1*0602) with susceptibility to MS (Oksenberg et al., 2008). The DR15 haplotype might contribute to MS risk by being involved in higher HLA molecule expression on APCs, incomplete negative selection of autoreactive CD4 T cells in the thymus, and preferential presentation of self-antigens to autoreactive T cells, rather promiscuous restriction of some potentially autoreactive CD4 T cell TCRs (recognition of identical peptides by one TCR in context of all MS-associated DR15 haplotypes) (Prat et al., 2005; Sospedra and Martin, 2005; Sospedra et al., 2006; Martin et al., 2021). In addition, environmental factors, such as virus infections, are suspected to contribute to MS origin. Several studies reported an increased risk for MS after EBV infection and clonal expansion of EBV-specific T cells in MS patients (Lünemann et al., 2008; Handel et al., 2010). Potential contributions of EBV infections to MS pathogenesis include priming of T cells with EBV-derived epitopes and re-activation by CNS autoantigens, or EBV-dependent activation and maintenance of an autoreactive B cell pool that is less susceptible to peripheral tolerance and that might serve as APCs (Lünemann et al., 2007, 2008; Wang J. et al., 2020). It has been proposed that viral epitopes that are structurally similar to myelin-derived self-peptides stimulate autoreactive T cells *via* molecular mimicry (Figure 2C; Wucherpfennig and Strominger, 1995; Wang J. et al., 2020).

Also B cells have a crucial role in MS disease progression. Clonally expanded autoreactive B cells are present in the brain parenchyma, meninges, and CSF and produce characteristic antibodies which are detectable in the CSF as oligoclonal bands (Dendrou et al., 2015). Memory B cells act as APCs and promote auto-proliferation of autoreactive CD4 T cells *via* presentation of CNS self-antigens, therefore shaping an autoreactive T cell repertoire in MS (Jelcic et al., 2018; Wang J. et al., 2020). In addition, CD20+ B cell depletion therapies ameliorate MS symptoms since they deplete most B cell subpopulations, only sparing antibody-producing plasma cells, which mostly do not express CD20 and are not professional antigen presenters. Thus, depletion therapies might function by blocking B cell antigen presentation to autoreactive T cells and releasing pro-inflammatory cytokines (Hauser et al., 2008; Barr et al., 2012; Dendrou et al., 2015). These studies emphasize the crucial role of T and B cell reactions and autoantigens in MS pathology.

### Narcolepsy

Narcolepsy is a rare neurological disorder caused by an autoimmune attack of T cells against self-antigens of hypocretin-producing neurons, leading to neuronal loss in the lateral hypothalamus (Bassetti et al., 2019). Polymorphisms in T cell function-related genes, such as T cell receptor alpha locus, were significantly associated with narcolepsy (Hallmayer et al., 2009). In 2018, Latorre and colleagues reported that CD8 and CD4 T cells target antigens of hypocretin neurons in narcolepsy patients and linked for the first time CD4 T cell autoreactivity to the pathophysiology of narcolepsy (Figure 2D and Table 1; Latorre et al., 2018). Earlier observations indicated a link between H1N1 influenza and narcolepsy, as the incidence of narcolepsy increased during 2009/10 pandemic and vaccination campaign with Pandemrix vaccine (Partinen et al., 2014). This prompted the hypothesis that narcolepsy may be an autoimmune disorder, potentially caused by molecular mimicry between epitopes on influenza antigens and hypocretin. The matter is still under investigation, but Latorre and colleagues have shown that hypocretin-specific CD4 T cells did not cross-react with influenza vaccine-derived antigens, thus excluding this kind of molecular mimicry (Latorre et al., 2018). However, another group found cross-reactivity between hypocretin-derived antigens and an epitope of the hemagglutinin flu protein of the pandemic 2009 H1H1 strain (Luo et al., 2018). Potential mechanisms explaining HLA-DR-restricted response in narcolepsy are epitope spreading and antigen presentation of epitopes released by dying neurons and processed by extracellular proteases (Latorre et al., 2018). In addition, it has been shown that antigen-specific CD8 T cells can destroy hypocretin neurons and trigger narcolepsy symptoms in mice (Bernard-Valnet et al., 2016). An immunophenotyping study further emphasizes the importance of T cells in narcolepsy *via* mass cytometry, revealing an immune cell signature in patients with narcolepsy, characterized by immune activation of T cells and increased production of pro-inflammatory cytokines and cytokines involved in B cell differentiation (Hartmann et al., 2016).

## T Cell Antigen-Specificity and Epitope Mapping Strategies

### The Missing Link Between Central Nervous System Disorders and Adaptive Immune Responses: Autoantigen Targets

So far, efforts have been made to link actions of the adaptive immune system with CNS disorders, as summarized above. In many cases, however, it is unclear whether the underlying T or B cell response is the cause or consequence of the associated neurologic/neurodegenerative disease, and further mechanistic details are often unknown.

Various studies associated changes in the composition of peripheral and CSF immune cell populations with neurodegenerative disease stages, thus linking particular adaptive immune cell signatures with the respective diseases (Saunders et al., 2012; Hartmann et al., 2016; Galli et al., 2019; Gericke et al., 2021; Wang P. et al., 2021). Progress in single-cell immunophenotyping techniques allows to measure the expression of multiple proteins or genes in individual cells *via* multi-color flow and mass cytometry, or single-cell RNA sequencing (scRNA-seq), respectively. Algorithm-guided analysis of high-dimensional single-cell data allows for characterization of immune cell subsets and population heterogeneity (Papalexi and Satija, 2018). Approaches to unravel the mechanistic origin of these descriptive association studies have led to an understanding of what type of adaptive immune cells might gain access to the CNS at certain entry sites and where their area of action lies. For example, lymphocyte infiltration to the brain in MS, PD, and AD appears to be a targeted process rather than passive extravasation due to BBB dysfunction, as mentioned earlier (Brochard et al., 2009; Ferretti et al., 2016; Laurent et al., 2017; Merlini et al., 2018). However, the antigenic target, the epitope presented by APCs in secondary lymphoid organs and at CNS borders for priming and re-activation of adaptive immune cells, often remains elusive.

The reasons for this are due to the nature of the main pathological features of the diseases. In neurological disorders such as narcolepsy and MS, detrimental autoreactive T cell responses target self-antigens directly deriving from neurons and their myelin sheaths, respectively. In AD, PD, and other neurodegenerative diseases, potential adaptive immune responses, whether harmful or beneficial, would have to be directed against aggregates of misfolded self-peptides such as Aβ, tau, and α-synuclein. While B cells can detect the 3D structure of aberrantly aggregated protein structures, T cells would have to respond to a linear epitope deriving from the self-peptide content of these aggregates (Sevigny et al., 2016; Arndt et al., 2018). Since most autoreactive T cells are eliminated by clonal depletion or tolerance formation in the thymus, self-reactive T cells would have to escape central tolerance, e.g., by showing only low-avidity interactions with APCs and the presented self-antigen (Klein et al., 2014; Koehli et al., 2014). Also, peripheral tolerance mechanisms initiated by Tregs would have to be circumvented to trigger an active autoimmune response (e.g., in MS) (Goverman, 2011).

### Detection of Self-Reactive T Cells

Therefore, the detection of autoreactive T cells and their corresponding self-antigen is challenging. Already within the normal, non-self-detecting, positively selected T cell repertoire, TCR affinity toward foreign antigens ranges only from *K*_d_ = 0.1–100 μM (compared to antibody *K*_d_ usually in nM range due to affinity maturation) (Davis et al., 1998). Autoreactive T cells that escaped negative selection typically have an affinity of *K*_d_ > 10 μM (Koehli et al., 2014). Frequencies of antigen-specific T cells within the memory repertoire strongly vary depending on the status of the immune response. Usually, they range from 10^–5^ to 5%, with autoreactive T cells ranging from 10^–5^ to 10^–4^% (Bacher et al., 2013). Thus, detection of autoreactive T cells requires sensitive methods.

Traditionally, the interaction between TCR and p-MHC complex is assessed *via* the functional response of T cells by measuring cell proliferation, cytolytic activity, expression of activation markers, and cytokine production. Various reports described measurement of cell proliferation *via* CFSE or H^3^ thymidine to identify Aβ-specific T cells in AD (Monsonego et al., 2003; Dhanwani et al., 2020). Moreover, cytokine production *via* ELISPOT has been employed to identify autoreactive T cells in PD (Sulzer et al., 2017). Some strategies have been developed to circumvent the low-frequency issue of autoreactive T cells in peripheral blood, including peptide pulsing and cell population expansion techniques. Notably, the T cell library method allowed the detection of rare antigen-specific T cells in narcolepsy by generating a library of polyclonally expanded T cells (Geiger et al., 2009; Latorre et al., 2018). Alternatively, antigen-specific T cells are detected with soluble fluorescently labeled p-MHC tetramers (Dolton et al., 2015). However, the number of peptide candidates is limited, p-MHC complexes are donor-specific and require prior knowledge of the donor’s haplotype. Moreover, due to the low avidity of TCRs toward p-MHC:autoantigen complexes, tetramer-based assays are not suitable for autoreactive CD4 T cells (Sospedra and Martin, 2005). All these approaches assume prior knowledge of epitope candidates. On the other hand, TCR-directed methods screen T cells against a large library of peptide epitopes. For instance, a decapeptide library containing more than four trillion sequences allowed the identification of GDP-L-fucose and RASGRP2 autoantigens in MS (Pinilla et al., 1994; Planas et al., 2018; Wang J. et al., 2020). However, for all autoreactive T cell clones that have been identified by the abovementioned techniques, the question is how their low numbers, low avidity toward APCs and antigen, and exposure to Tregs in the periphery impact their relevance for the respective disease.

### Influence of Infectious Diseases and Molecular Mimicry

Many autoimmune responses are influenced by environmental factors such as concomitant infections in addition to a risk-increasing immunogenetic background. Some expanded T cell clones discovered in neurological/neurodegenerative diseases revealed responses against viral antigens (Lünemann et al., 2008; Luo et al., 2018; Gate et al., 2020; Wang J. et al., 2020). These infections can contribute to completely different, potentially autoreactive immune responses in different ways. On the one hand, they might drive or exacerbate autoimmune responses by creating an enhanced pro-inflammatory environment. Consequently, increased numbers of activated immune cells might be attracted to the site of infection. These cells may include APCs with increased expression of co-stimulatory factors, which facilitates local antigen presentation. On the other hand, they could interfere with peripheral tolerance mechanisms, facilitating the action of previously suppressed autoreactive effector cells. Moreover, considering that aging is a significant risk factor for many neurodegenerative diseases, acute or chronic viral infections in later life might also mask CNS disease-specific adaptive immune responses. For example, it has been suggested that age-associated changes in CD8 T cell populations may be caused by chronic CMV-mediated antigenic stimulation (Chidrawar et al., 2009; Derhovanessian et al., 2010). Only a few studies have controlled for the effect of *Herpesviridae* infections on T cell abundance in neurodegenerative diseases such as AD, and often the effect of background viral infections is neglected (Pellicanò et al., 2012; Gericke et al., 2021). Apart from bystander activations of innate (APCs) and adaptive immune responses, infectious diseases might also be the trigger for the development of an autoreactive immune response through molecular mimicry between peptides derived from infectious agents and self-proteins (Sospedra and Martin, 2006). Antigen cross-recognition by both T cells and antibodies is a highly necessary mechanism to cover the large number of possible foreign antigens, considering that the negatively selected TCR repertoire is smaller than the amount of potentially occurring antigens (Mason, 1998; Borbulevych et al., 2009; Macdonald et al., 2009). Cross-recognition does not rely on perfect homology of epitope sequences, but in fact, works when peptides share common amino acid motifs or have only similarly charged amino acids in the same position (Hemmer et al., 1998). In addition, cross-restriction, the ability of one TCR to detect an antigen on different HLA haplotypes, further facilitates potential molecular mimicry between, for example, viral epitopes and self-peptides (Yousef et al., 2012).

### Peptide Splicing and the Generation of Neo-Epitopes

All the abovementioned mechanisms rely on pre-existing sets of linear peptide epitopes that can be detected by potentially autoreactive T cells. However, these T cells would always be subject to clonal depletion or tolerance mechanisms and therefore only present in small numbers. Over the last two decades, the concept of T cell neo-epitopes, against which there is tolerance in only a few cases, has increasingly emerged. In B cell research, it has been long known that aggregation of self-proteins increases immunogenicity due to novel three-dimensional targets. Since T cells bind to linear peptide epitopes with ideally nine amino acid core regions, the only way to create neo-epitopes is *via* ‘splicing’ or ‘fusion’ of dis-continuous peptide segments from one or two different proteins (Faridi et al., 2021). Most variants of spliced neo-epitopes have been observed for HLA type I-restricted antigens from cancer research (Hanada et al., 2004; Dalet et al., 2011; Vigneron et al., 2017). Following partial proteolysis in the proteasome and formation of short peptide segments, transpeptidation events would give rise to novel linear peptide sequences with potential immunogenicity (Mishto et al., 2012). The discovery of CD4 T cell clones from type I diabetic patients recognizing a so-called ‘trans-spliced’ peptide which resulted from splicing between two different beta cell autoantigens confirmed the existence of HLA type II-restricted neo-epitopes (Delong et al., 2016). These unpredictable kinds of neo-epitopes could be retrieved *ex vivo/in vitro* by purification of loaded HLA-peptide complexes from the surfaces of APCs and analysis of the bound peptides by liquid chromatography-tandem mass spectrometry (Purcell et al., 2019). However, the lack of sequence information for spliced peptides in the predicted proteome would again complicate the interpretation of this data (Faridi et al., 2021).

### Computational Strategies for T Cell Epitope Discovery

Alternative techniques for epitope mapping are needed since the abovementioned mechanisms of cross-presentation/molecular mimicry, and peptide splicing make it difficult to generate epitope libraries against which a discovered clonally expanded T cell clone from a particular disease background might react. The limitations of current approaches prompted the development of computational methods to predict T cell epitopes. Several groups developed *in silico* tools to predict peptide binding to multiple MHC haplotype alleles, the most renowned being NetMHCpan (Jurtz et al., 2017; Peters et al., 2020). The high performance of NetMHCpan is conferred by training a neural network using both binding affinities of peptides to MHC molecules and elution data of naturally processed and presented ligands (Jurtz et al., 2017; Peters et al., 2020). However, a drawback of MHC binding prediction is the inability to accurately predict T cell immunogenicity (Peters et al., 2020; Joglekar and Li, 2021). Prediction of MHC binding allows to limit the number of potential T cell epitopes to screen, as it was done in several studies (Newell et al., 2013; Cassotta et al., 2019; Saini et al., 2021). A common approach to identify functionally relevant TCRs is investigation of identical TCRs (e.g., clonally expanded or ‘public’ TCRs). However, TCR repertoires are extremely diverse and the presence of shared TCRs within or between individuals is rare (Mayer-Blackwell et al., 2021). Recently developed computational approaches that predict the target antigen of TCRs imply that similar complementarity-determining regions (CDRs) share antigen specificity (Dash et al., 2017; Glanville et al., 2017). Two algorithms, GLIPH and TCRdist, showed for the first time that TCRs recognizing the same antigen share sequence and structural features and can be assigned to clusters to predict their target antigens (Dash et al., 2017; Glanville et al., 2017; Huang et al., 2020; Mayer-Blackwell et al., 2021). Other tools were later developed to cluster TCRs from bulk-sequenced repertoires, including ALICE, TCRNET and RepAN (Ritvo et al., 2018; Pogorelyy et al., 2019; Yohannes et al., 2021). Recent advances in scRNA-seq technologies allow to obtain matching gene expression and TCR sequences at the single-cell level. This sparked the development of algorithms that predict antigen specificity of single cells using machine learning models on known TCR-antigen pairs (Fischer et al., 2020; Chronister et al., 2021; Pai and Satpathy, 2021). However, these algorithms are unable to achieve high prediction accuracy on the entire TCR repertoire (Pai and Satpathy, 2021). In parallel, CoNGA, the first algorithm that enables large-scale characterization of relationships between T cell antigen specificities and phenotypes was developed, allowing a deeper understanding of T cell biology (Schattgen et al., 2021). Moreover, the first effort of combining spatial transcriptomics and TCR sequencing was attempted in the context of human brain metastasis (Hudson and Sudmeier, 2021). Overall, scRNA-seq and TCR profiling have a huge potential to transform the field of computational immune prediction and epitope mapping.

## Concluding Remarks and Perspective

The immune system has a pivotal role in CNS development and maintenance in the steady state by ensuring immune surveillance, regulated traffic of immune cells, and drainage of CNS antigens into peripheral lymph nodes. The functional decline of adaptive immune responses and deterioration of CNS clearance systems during aging and neurodegeneration leads to infiltration of immune cells in the brain. In addition, the critical role of adaptive immune responses in neurodegeneration diseases is well established. However, there are still many unanswered questions about antigen specificity of CNS-infiltrating, potentially autoreactive T cells. Paired single-cell transcriptomics and TCR sequencing technologies may allow high-throughput epitope mapping. T cell epitope prediction strategies are currently used for the development of epitope-specific vaccinations and adoptive immunotherapies for virus infections and cancer (Provenzano et al., 2006; Gubin et al., 2015; Stryhn et al., 2020; Berzero et al., 2021). TCR epitope-mapping technologies may potentially be used for diagnosis and prediction of immune responses, as shown by the examples of cutaneous T cell lymphoma and celiac disease, viral infections, and tumor neoantigens (Kirsch et al., 2015; Kato et al., 2018; Wolf et al., 2018; Ahmadzadeh et al., 2019; Chiou et al., 2021; Yao et al., 2021).

## Author Contributions

CR contributed to the conceptualization, writing – original draft, visualization, and funding acquisition. CG contributed to the conceptualization, writing – review and editing, supervision, and funding acquisition. Both authors contributed to the article and approved the submitted version.

## Conflict of Interest

The authors declare that the research was conducted in the absence of any commercial or financial relationships that could be construed as a potential conflict of interest.

## Publisher’s Note

All claims expressed in this article are solely those of the authors and do not necessarily represent those of their affiliated organizations, or those of the publisher, the editors and the reviewers. Any product that may be evaluated in this article, or claim that may be made by its manufacturer, is not guaranteed or endorsed by the publisher.
